# Casticin Induces DNA Damage and Affects DNA Repair Associated Protein Expression in Human Lung Cancer A549 Cells (Running Title: Casticin Induces DNA Damage in Lung Cancer Cells)

**DOI:** 10.3390/molecules25020341

**Published:** 2020-01-15

**Authors:** Zheng-Yu Cheng, Yung-Ting Hsiao, Yi-Ping Huang, Shu-Fen Peng, Wen-Wen Huang, Kuo-Ching Liu, Te-Chun Hsia, Tzong-Der Way, Jing-Gung Chung

**Affiliations:** 1 Department of Biological Science and Technology, China Medical University, Taichung 404, Taiwan; kaller2826@gmail.com (Z.-Y.C.); u102301851@cmu.edu.tw (Y.-T.H.); t20811@mail.cmuh.org.tw (S.-F.P.); wwhuang@mail.cmu.edu.tw (W.-W.H.); 2 Department of Physiology, College of Medicine, China Medical University, Taichung 404, Taiwan; yphuang@mail.cmu.edu.tw; 3 Department of Medical Research, China Medical University Hospital, Taichung 404, Taiwan; 4 Department of Medical Laboratory Science and Biotechnology, China Medical University, Taichung 404, Taiwan; kchliu@mail.cmu.edu.tw; 5 Department of Respiratory Therapy, China Medical University, Taichung 404, Taiwan; D1914@mail.cmuh.org.tw; 6 Department of Internal Medicine, China Medical University Hospital, Taichung 404, Taiwan

**Keywords:** casticin, human lung cancer A549 cells, DNA damage, DNA condensation and repair

## Abstract

Casticin was obtained from natural plants, and it has been shown to exert biological functions; however, no report concerns the induction of DNA damage and repair in human lung cancer cells. The objective of this study was to investigate the effects and molecular mechanism of casticin on DNA damage and repair in human lung cancer A549 cells. Cell viability was determined by flow cytometric assay. The DNA damage was evaluated by 4’,6-diamidino-2-phenylindole (DAPI) staining and electrophoresis which included comet assay and DNA gel electrophoresis. The protein levels associated with DNA damage and repair were analyzed by western blotting. The expression and translocation of p-H2A.X were observed by confocal laser microscopy. Casticin reduced total viable cell number and induced DNA condensation, fragmentation, and damage in A549 cells. Furthermore, casticin increased p-ATM at 6 h and increased p-ATR and BRCA1 at 6–24 h treatment but decreased p-ATM at 24–48 h, as well as decreased p-ATR and BRCA1 at 48 h. Furthermore, casticin decreased p-p53 at 6–24 h but increased at 48 h. Casticin increased p-H2A.X and MDC1 at 6–48 h treatment. In addition, casticin increased PARP (cleavage) at 6, 24, and 48 h treatment, DNA-PKcs and MGMT at 48 h in A549 cells. Casticin induced the expressions and nuclear translocation of p-H2AX in A549 cells by confocal laser microscopy. Casticin reduced cell number through DNA damage and condensation in human lung cancer A549 cells.

## 1. Introduction

Lung cancer is the single most significant cause of cancer mortality worldwide [1], and it causes approximately 1.5 million deaths globally per year [2,3,4,5,6]. Lung cancer is classified histologically into two types: small cell lung cancer (SCLC) and non-small cell lung cancer (NSCLC) [2,5,7,8]. The overall 5-year survival rate of lung cancer, including SCLC and NSCLC, is the lowest (18%) among all cancers [9]. In Taiwan, lung cancer is also the leading cancer death, where about 39.2 individuals per 100,000 die annually from lung cancer according to the report of the Department of Health, R.O.C. (Taiwan) in 2016 [10]. Currently, it is an attractive and potential strategy to find new agents from herbal plants for lung cancer patients.

Anticancer compounds induced DNA damage, including single-strand break (SSB) lesions or double-strand breaks (DSBs) of DNA, and resulted in replication errors of genomic DNA, genomic instability, and cell death in cancer cells [11,12]. Eukaryotic cells have evolved DNA damage responses (DDR) to reply and signal the DNA damage [11,13,14]. Furthermore, numerous evidence also demonstrated that DNA damage is a critical factor for examining carcinogen compounds [15,16]. The ability to induce cancer cell DNA damage will be a critical examining factor for clinically anticancer drugs in vitro and in vivo.

Casticin, an active flavonoid, was isolated from a Chinese herb Vitex Fructus [17] which has been used to treat inflammation in China population. Casticin was documented to exert wide spectrums of biological and pharmacological activities, including immunomodulatory [18], anti-hyperprolactinemia [19], neuroprotective [20], and anti-tumor [21,22]. It also suppressed the eosinophil migration and chemokine levels in A549 cells [23]. Furthermore, casticin may have the aptitude to treat inflammatory diseases of lungs for suppressing lipopolysaccharide (LPS)-induced lung injury via inhibiting TNF-α, IL-6, and IL-1β (inflammatory cytokines) [24,25]. Besides, casticin enhanced the effects of chemotherapeutic drugs for cancer therapy [26].

In our earlier in vitro studies, we demonstrated that casticin triggered DNA damage in B16F10 melanoma cells [27]. However, the effects of casticin on DNA damage and repair in human NSCLC A549 cells is still uncertain. Thus, in this study, we attempt to investigate the effects and molecular mechanism of casticin on DNA damage and repair in human lung cancer A549 cells and found that casticin induced DNA damage and down-regulated the expressions of DNA repair-related proteins.

## 2. Results

### 2.1. Casticin Decreased Viable Cell Number of A549 Cells

**Figure 1:**
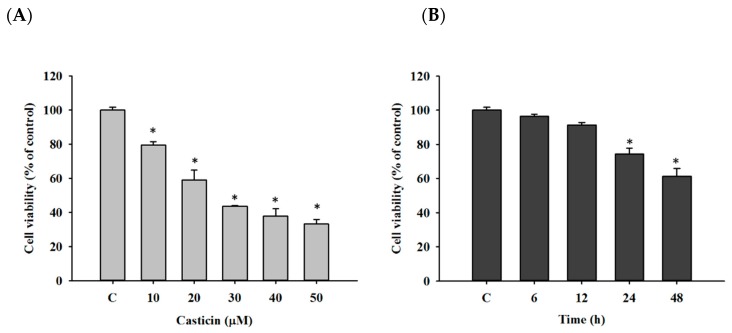
Casticin decreased the percentage of viable cells in A549 cells. Cells were incubated with 0, 10, 20, 30, 40, and 50 μM of casticin for 48 h (**A**) or treated with 20 μM of casticin for 0, 6, 12, 24, and 48 h (**B**) and then were collected for measuring the percentage of viable cells by flow cytometry. Experiments were performed in triplicate as described in Materials and Methods. Data represent mean ± S.D. * *p* < 0.05 was significant difference between casticin-treated and control groups.

At first, cell viability was evaluated by flow cytometric assay. A549 cells were treated with different doses of casticin (0,10, 20, 30, 40, and 50 μM) for 48 h, or cells were incubated with 20 μM of casticin for defined periods (0, 6, 12, 24, and 48 h). After treatment, individual cells were trypsinized and harvested for quantifying total viable cell numbers by propidium iodide (PI) exclusion assay. As shown in Figure 1, the increase of casticin led to the reduction of viable cell numbers after 48 h incubation, and these effects are concentration-dependent (Figure 1A). Furthermore, the long-time treatment of casticin resulted in the lower viable cell number, and these effects are time-dependent (Figure 1B).

### 2.2. Casticin Induced Chromatin Condensation in A549 Cells

**Figure 2:**
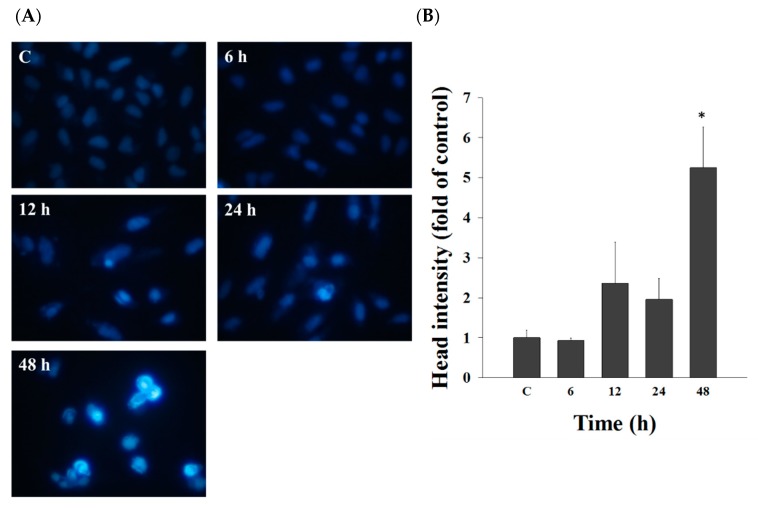
Casticin affected DNA condensation in A549 cells. Cells (1 × 10^5^ cells/well) were grown in 12-well plates for 24 h and incubated with 20 μM of casticin for 0, 6, 12, 24, and 48 h. Cells were fixed with 3.7% paraformaldehyde (*v*/*v*) in phosphate buffered saline (PBS) for 15 min, permeablized with 0.1% Triton X-100 in PBS for 5 min. Nuclei were stained with 2 μg/mL of 4′,6-diamidino-2-phenylindole (DAPI) for 10 min. All samples were examined and photographed using a fluorescence microscope at 200× (**A**) and were measured the intensity of fluorescence (**B**) as described in Materials and Methods. Data represent mean ± S.D. * *p* < 0.05 was significant difference between casticin-treated and control groups.

To investigate chromatin condensation, we treated A549 cells with casticin (20 μM) for different times, and cells were stained with DAPI. In Figure 2, casticin at 12–48 h treatment significantly caused chromatin condensation, displaying the lighter DAPI staining (Figure 2A) and higher fluorescent intensity (Figure 2B) than that in control groups in A549 cells.

### 2.3. Casticin Induced DNA Damage in A549 Cells

**Figure 3:**
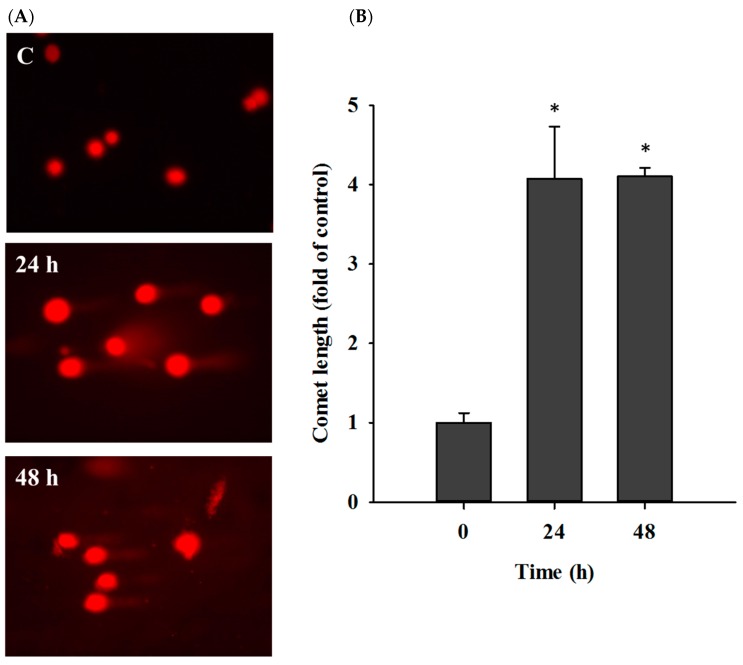
Casticin induced DNA damage in A549 cells. Cells were incubated with 20 μM of casticin for 24 and 48 h and analyzed by Comet assay (**A**) and then calculated the fluorescence intensity of comet (**B**) as described in Materials and Methods. Data represent mean ± S.D. * *p* < 0.05 was significant difference between casticin-treated and control groups.

For understanding the reduction of total cell viability in casticin-treated A549 whether or not via the induction of DNA damage, cells were treated with casticin (20 μM) for 24 and 48 h, and then the DNA damage was determined by comet assay (Figure 3). Results indicated that casticin significantly induced DNA damage at 24 and 48 h treatment, resulting in the development of comet tails in A549 cells.

**Figure 4:**
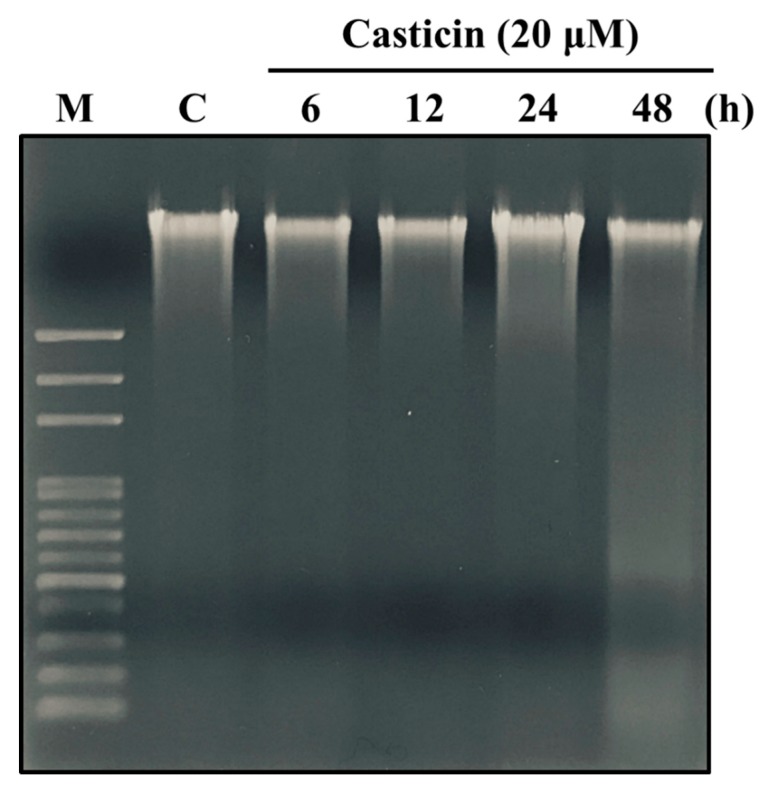
Casticin induced DNA fragmentation in A549 cells. Cells were incubated with 20 μM of casticin for 0, 6, 12, 24, and 48 h. Then cells were collected and lysed and individual DNA was extracted for DNA gel electrophoresis as described in Materials and Methods.

DNA damage of A549 cells treated with casticin was assessed by DNA gel electrophoresis. Cells were exposed to 20 μM of casticin for various periods, and individual DNA was isolated and electrophoresed on an agarose gel (Figure 4). Results showed that casticin triggered DNA damage (smeared DNA) at 48 h treatment, indicating the development of DNA damage.

### 2.4. Casticin Affected the Levels of DNA Damage-Associated Proteins in A549 Cells

**Figure 5:**
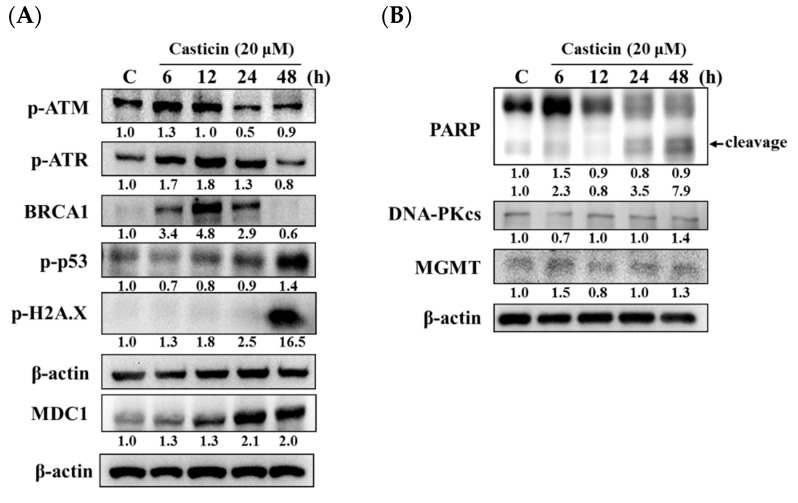
Casticin affects the DNA damage and repair associated protein expressions in A549 cells. Cells were incubated with 20 μM casticin for 0, 6, 12, 24, and 48 h, the cells were collected for western blotting, and the resultant membranes were used to probe to anti-p-ATM, -p-ATR, -BRCA1, -p-p53, -p-H2A.X, -MDC1 (**A**) -PARP, -DNA-PKcs, and -MGMT (**B**) as described in Materials and Methods. β-actin was used as an internal control.

The effects of casticin on the levels of DNA damage-associated proteins were investigated by western blotting. A549 cells were treated with casticin (20 μM) for defined times (0, 6, 12, 24, and 48 h), and then cells were harvested for western blotting assay. As shown in Figure 5, casticin increased p-ATM at 6 h and decreased at 24–48 h treatment, p-ATR and BRCA1 increased at 6–24 h treatment but reduced at 48 h (Figure 5A). Furthermore, casticin decreased p-p53 at 6–24 h but increased at 48 h. Casticin increased p-H2A.X at 6–48 h and increased MDC1 at 6-48 h treatment, and these effects are time-dependent. In addition, casticin increased PARP (cleavage) at 6, 24, and 48 h, DNA-PKcs, and MGMT at 48 h treatment in A549 cells (Figure 5B).

### 2.5. Casticin Affected the Expression and Translocation of p-H2AX on A549 Cells

**Figure 6:**
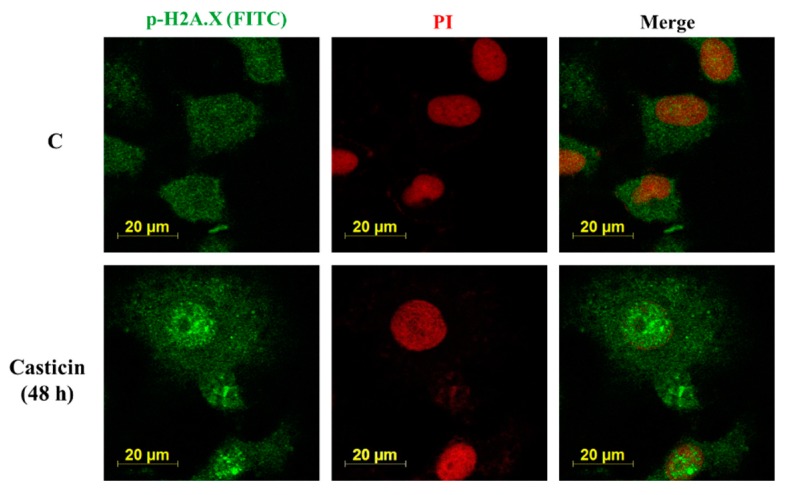
Casticin affected the translocation of p-H2A.X in A549 cells. Cells (5 × 10^4^ cells/well) were plated on 4-well chamber slides and incubated with 0 and 20 μM of casticin for 48 h, and cells were stained by anti-p-H2A.X (green fluorescence) and then stained with secondary antibody fluorescein isothiocyanate (FITC-conjugated goat anti-mouse IgG). All cells were counterstained by propidium iodide (PI) (red fluorescence) for nucleus examination and were photomicrographed under a Leica TCS SP2 Confocal Spectral Microscope as described in Materials and Methods.

Casticin affected p-H2A.X protein expression in A549 cells by western blotting. Thus, for further confirming the expression and translocation of p-H2A.X were induced by casticin, A549 cells were incubated with casticin (20 μM) for 48 h and then observed and photographed under confocal laser microscopy. As shown in Figure 6, casticin promoted the level and translocation of p-H2A.X in nucleus at 48 h treatment in A549 cells.

## 3. Discussion

Anticancer drugs, derived from natural products, decrease cancer cell numbers by inducing cell apoptosis in vitro or by suppressing the tumor growth in xenografted animal models in vivo [28,29,30]. In addition, some anticancer compounds induced DNA damage and halted DNA repair associated protein in cancer cells, eventually leading to cancer cell death. Therefore, the effects and molecular mechanism of casticin on the induction of DNA damage of A549 cells were investigated in vitro, based on the fact that casticin has been shown to trigger cell death apoptosis in various human lung cancer cell lines in vitro [31,32,33].

The information regarding clinical cancer patients treated with casticin in the future is not clear yet. Casticin is isolated from the mature and dried fruits of *Vitex trifolia* L. and the fruits have been commonly used as a folk medicine for treatment of headache, cold, migraine, and eye pain in China [34]. It is used for the treatment of premenstrual syndrome (PMS) in European with little adverse effects [19]. Casticin displayed genotoxic or cytotoxic effects on cancer cells at low doses and on normal cells at high doses [31,35]. Recently, casticin was documented to exert potential anti-cancer effects, but the limitation of casticin may be the poor water solubility and the fact that few pharmacokinetic studies are available [36].

Casticin suppressed the total viable cell number of A549 cells in a dose-dependent manner (Figure 1), and the result is consistent with other reports. DNA condensation and damage, leading to cell death, were involved in reducing A549 cell viability [32]. Both DAPI staining and comet assays were employed for examining chromatin condensation and DNA damage [37,38]; thus, in this study, we utilized both approaches to evaluate chromatin condensation and DNA damage in A549 cells after exposure to casticin, respectively. As shown in Figure 2, casticin caused chromatin condensation in a time-dependent manner, which is consistent with other anticancer drugs approved by using DAPI staining [39,40]. Plasticity of chromatin structure is involved in cellular response to DNA damage [41,42] because it is the physiological template for DNA repair machinery for maintaining and restoring genome integrity [43]. Furthermore, DNA electrophoresis was utilized to survey DNA damage [40,44]. Herein, we also found that casticin induced the presence of laddered DNA patterns, indicating the DNA fragmentation in A549 cells in vitro that is in agreement with another report [45].

For confirming that casticin reduced total viable cell numbers were related to the protein level of DNA damage and repair in A549 cells, cells were thus grown in medium containing 20 μM of casticin for various periods, and cell proteins were collected and assayed by western blotting. As shown in Figure 5A, casticin increased phospho-ataxia telangiectasia mutated (p-ATM) at 6 h treatment and decreased at 24–48 h treatment, as well as increased phospho-ataxia telangiectasia and rad3-related (p-ATR) and BRCA1 at 6-24 h but decreased at 48 h treatment. But casticin decreased p-ATM, p-ATR, and BRCA1 at 48 h treatment in A549 cells. The p-p53 was decreased at 6–24 h and increased at 48 h treatment, but p-H2A.X and MDC1 was increased from 6–48 h, and these effects are time-dependent, thus suggesting that the responses of DNA damage are effective in A549 cells. However, casticin increased PARP (cleavage), DNA-PKcs, and MGMT at 24–48 h, 48 h, and 48 h, respectively (Figure 5B). It is well-documented that DNA damage occurs, and then p53 will accumulate [46]. Furthermore, DNA breaks induce the phosphorylation of a multitude of substrates and leads to activation of ATM, ATR, and DNA-PKcs, which are members of the PIKK (phosphatidylinositol 3-kinase-like kinase) family, and allows them to arrange DNA repair and cell recovery [47]. After DNA damage, p53 is phosphorylated by ATM for the stabilization and transactivation of its target genes [48]. Furthermore, deficiency of ATM is involved in lymphoid malignancies in humans and mice [49,50,51].

Figure 5B also revealed that casticin augmented the active form of PARP at 24–48 h treatment, indicating that DNA damage developed at 6–12 h, and then cells started to repair DNA damage at 24–48 h. The synthesis of PARP-1 is linked to DNA repair in the nucleus, and the activation of PARP-1 led to an intrinsic apoptosis for leading to the decrease of viable cell numbers [52]. Herein, we also found that casticin increased the level of MDC1 in A549 cells at 6–48 h treatment. PARP1 detects and binds to single-strand DNA breaks (SSBs) and responds by initiating the repair of these lesions. The protein expressions of the DNA double-strand breaks (DSBs) pathway and the inhibition of PARP1 can lead to inducing the formation of SSBs [53]. The MRN/ATM interacts with at the site of DNA breaks, which augments ATM’s kinase activity and phosphorylates the histone H2A.X at Ser 139, and results in γH2A.X, which is the first step in the recruitment of MDC1, 53BP1, and BRCA1 [47] to facilitate DNA repair [54]. The occurrence of DSB is also strongly dependent upon chromatin structure and ATM kinase phosphorylation, one of the several downstream targets, such as H2A.X [55]; furthermore, the phosphorylated H2A.X initiates a cascade of repair factor recruitment [11,14]. Comparing human A549 and mouse B16F10 cells after treatment with casticin, the expressions of MGMT, MDC1, DNA-PK, and p-ATM were different due to the differences of their genetic background, while the former is KRAS mutant, the later wild type [56,57,58].

Different mechanisms were involved in casticin-induced responses that have been reported in many human cancer cells in vitro. Casticin induced cytotoxicity and reduced cell proliferation by blocking the phosphorylation of Akt (Ser473) and mTOR (Ser2448) proteins, indicating the involvement of Akt/mTOR signaling pathway, in human breast cancer MCF-7, gastric cancer SNU16, and myeloma RPMI 8226 cells [26]. Besides, casticin decreased lung cancer stem-like cell characteristics of H446 cells and induced apoptosis by activating FoxO3a [33]. FOXO3a, a transcription factor, is involved in various cellular processes, including cell cycle arrest, DNA repair, and tumor suppression [59]. Morever, in cancer progression, FOXO3a overexpression suppressing cancer cell growth, induces apoptosis, and reduces tumor size [60]. Casticin promoted FOXO3a dephosphorylation and FOXM1 inactivation, leading to growth suppression and cell cycle arrest in hepatocellular carcinoma cells [61]. Dephosphorylation of active FOXO3a induces cell cycle arrest and apoptosis [62]. Forkhead box M1 (FOXM1) is a downstream target of FOXO3a [63] and is frequently upregulated in cancer cells [64].

Furthermore, casticin exerts anti-inflammatory effects and it reduced the levels of IL-6, tumor necrosis factor α (TNF-α), IL-8, COX-2, and prostaglandin E2. It also reduced Mucin 5AC, a pro-inflammatory cytokine, and inhibited ICAM-1 expression for monocyte adhesion via suppression of the PI3K/Akt, NF-κB, and MAPK signaling pathways in IL-1β-stimulated inflammatory pulmonary epithelial cells [65].

**Figure 7:**
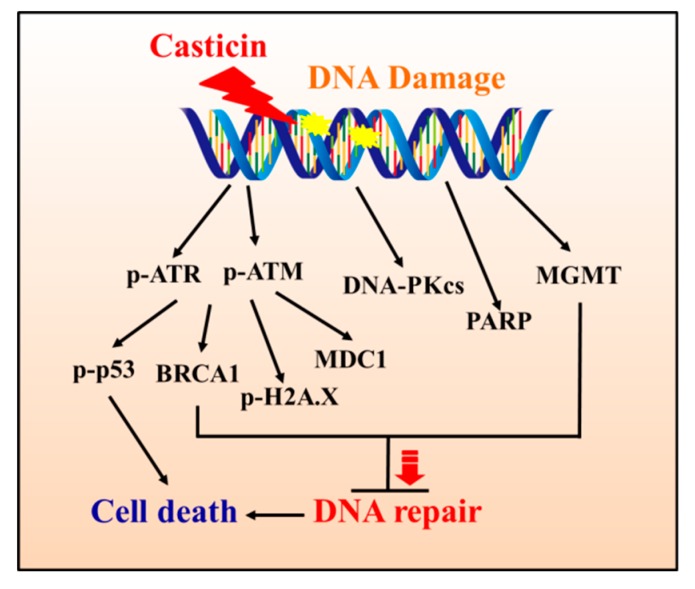
The possible mechanism of casticin-induced DNA repair and cell death in A549 cells.

Based on the above observations and findings, we concluded that casticin induced cytotoxic effects via the reduction of viable cell numbers and induction of DNA damage in A549 cells in vitro. The results of DAPI staining, comet assay, and the expressions of DNA damage-associated proteins, such as p-ATM, p-ATR, BRCA1, MDC1, H2A.X, p-p53, and MGMT, in A549 cells can provide the evidence for illustrating the involved mechanism, and the mechanism is summarized in Figure 7.

## 4. Material and methods

### 4.1. Chemicals and Reagents

Casticin, dimethyl sulfoxide (DMSO), propidium iodide (PI), 4’,6-diamidino-2-phenylindole (DAPI), and trypsin-EDTA were purchased from Sigma-Aldrich Corp. (St. Louis, MO, USA). RPMI-1640 medium, fetal bovine serum (FBS), L-glutamine, and antibiotics (penicillin and streptomycin) were obtained from Invitrogen Life Technologies (Carlsbad, CA, USA). Anti-MGMT, -PARP, -p-ATM, -p-ATR, and -β-actin were obtained from Calbiochem (San Diego, CA, USA), anti-p-H2A.X, and -BRCA1 from GeneTex Inc. (Irvine, CA, USA), p-p53 from Santa Cruz Biotechnology Inc. (Santa Cruz, CA, USA), and MDC1 from Millipore (Billerica, MA, USA). Secondary antibody (anti-mouse IgG) was purchased from Amersham Pharmacia Biotech, Inc. (Piscataway, NJ, USA).

### 4.2. Cell Culture

A549 cells (human lung cancer) were obtained from the Food Industry Research and Development Institute (Hsinchu, Taiwan). Cells were cultured in RPMI-1640 medium containing 10% heat-inactivated FBS, 2.0 g/L sodium bicarbonate, and antibiotics (100 units/mL penicillin and 100 μg/mL streptomycin) at 37 °C in a humidified incubator of 5% CO_2_ [66].

### 4.3. Measurements of Total Cell Viability

A549 cells were seeded in 12-well plates at a density of 2 × 10^5^ cells/well overnight and treated with adequate doses of casticin (0, 10, 20, 30, 40, and 50 μM) for 48 h. Or cells were exposed to 20 μM of casticin for defined periods (0, 6, 12, 24, and 48 h). After treatment, cells from individual treatments were trypsinized, collected, wash, and then re-suspended in PI solution (5 μg/mL) for determining cell viability using flow cytometer as cited previously [66].

### 4.4. DAPI Staining

A549 cells (2 × 10^5^ cells/well) were seeded in 12-well plates overnight and incubated with casticin (20 μM) for various periods (0, 6, 12, 24, and 48 h). After incubation, cells were treated with 4% paraformaldehyde in phosphate buffered saline (PBS) for 15 min, permeabilized by 0.1% Triton X-100 for 5 min, and stained with DAPI solution (2 μg/mL) for 10 min. Finally, the individual sample was observed and snapped using a fluorescence microscope at 200× as cited previously [27,67].

### 4.5. Comet Assay

The DNA damage was evaluated by comet assay (single-cell electrophoresis). In brief, A549 cells (2 × 10^5^ cells/well) were incubated with casticin (20 μM) for 0, 24, and 48 h. Cells from individual treatment were surveyed for their DNA damage. The single-cell comet was randomly captured at a constant depth of the agarose gel. The length of the comet tail was measured and quantified using Tri Tek Comet Score^TM^ software (TriTek Corp, Sumerduck, VA, USA) as described previously [27,66].

### 4.6. DNA Gel Electrophoresis

A549 cells (1 × 10^6^ cells/dish) were incubated with casticin (20 μM) for different times (0, 6, 12 24, and 48 h). After exposure, cells were trypsinized, harvested, and treated with DNA lysis buffer on ice for 30 min. DNA solution from individual treatments was isolated by centrifugation and then treated with proteinase K and RNase A. Finally, DNA was analyzed on 2% agarose gel, and the DNA patterns were photographed under UV-box as previously described [68,69].

### 4.7. Western Blotting

A549 cells (1 × 10^6^ cells/dish) were exposed to 20 μM of casticin for defined periods (0, 6, 12, 24, and 48 h). After treatment, cells were trypsinized and harvested. Total proteins were extracted by PRO-PREP™ Protein Extraction Solution from iNtRON Biotechnology, Inc. (Seoul, Korea). The extracted proteins were quantitated using BioRad assay kit. A defined amount of protein (30 μg) was loaded into 8–12% sodium dodecyl sulfate-polyacrylamide gel (SDS-PAGE), and the separated proteins were transferred onto polyvinylidene difluoride (PVDF) membranes (Millipore Corporation, Billerica, MA, USA). Subsequently, the membranes were blocked with 2.5% bovine serum albumin (BSA) for 1 h, probed with primary antibodies overnight at 4 °C, and then treated with the corresponding secondary antibodies conjugated with horseradish peroxidase for 1 h at room temperature. Finally, the signals of bound antibodies were examined by chemiluminescence kits (Merck KGaA, Darmstadt, Germany) as described previously [27,70].

### 4.8. Immunofluorescence Assay

Immunofluorescence assay was applied to detect the expression and translocation of p-H2A.X by confocal spectral microscopy. A549 cells (1 × 10^5^ cells/well) were grown on four-chamber slides and then treated with or without casticin (20 μM) for 48 h. After exposure, cells were treated with fixer (4% formaldehyde in PBS), permeabilized using 0.3% Triton-X 100, washed by PBS, probed with anti-p-H2A.X antibody, followed by fluorescein isothiocyanate (FITC)-conjugated secondary antibody (green fluorescence). Subsequently, cells were stained with the PI solution (red fluorescence) for nucleus staining, observed, and photographed under a Leica TCS SP2 confocal spectral microscopy as described previously [66,71].

### 4.9. Statistical Analysis

The data were presented as mean ± standard deviation (SD) and analyzed using one-way analysis of variance (ANOVA), and differences between the casticin-treated and -untreated (control) groups were considered statistically significant at the level of *p* < 0.05.
