# Erratum: Cheng, Z.Y., et al. Casticin Induces DNA Damage and Affects DNA Repair Associated Protein Expression in Human Lung Cancer A549 Cells (Running Title: Casticin Induces DNA Damage in Lung Cancer Cells). *Molecules* 2020, *25*, 341

**DOI:** 10.3390/molecules26020292

**Published:** 2021-01-08

**Authors:** Molecules Editorial Office

**Affiliations:** MDPI, St. Alban-Anlage 66, 4052 Basel, Switzerland; molecules@mdpi.com

We wish to report an error in the title of article [1]. The correct title of the article [1] is Casticin Induces DNA Damage and Affects DNA Repair Associated Protein Expression in Human Lung Cancer A549 Cells. We apologize for this error and state that the scientific conclusions are unaffected. The original article has been updated. 
